# Differential Recruitment of Dendritic Cells Subsets to Lymph Nodes Correlates with a Protective or Permissive T-Cell Response during* Leishmania* (*Viannia*)* Braziliensis* or* Leishmania* (*Leishmania*)* Amazonensis* Infection

**DOI:** 10.1155/2016/7068287

**Published:** 2016-03-17

**Authors:** A. K. Carvalho, K. Carvalho, L. F. D. Passero, M. G. T. Sousa, V. L. R. da Matta, C. M. C. Gomes, C. E. P. Corbett, G. E. Kallas, F. T. Silveira, M. D. Laurenti

**Affiliations:** ^1^Laboratory of Pathology of Infectious Diseases (LIM-50), Medical School, University of São Paulo, Avenida Dr. Arnaldo 455, 01246903 Cerqueira César, SP, Brazil; ^2^Laboratory of Immunology (LIM-60), Medical School, University of São Paulo, Avenida Dr. Arnaldo 455, 01246903 Cerqueira César, SP, Brazil; ^3^Laboratory of Leishmaniasis, Department of Parasitology, Evandro Chagas Institute, Surveillance Secretary of Health, Ministry of Health, Rodovia BR-316 Km 7 s/n, 67030-000 Ananindeua, PA, Brazil; ^4^Tropical Medicine Nucleus, Federal University of Pará, Avenida Generalíssimo Deodoro 92, 66055-240 Belém, PA, Brazil

## Abstract

*Leishmania (L.) amazonensis* (*La*) and* L. (V.) braziliensis* (*Lb*) are responsible for a large clinical and immunopathological spectrum in human disease; while* La* may be responsible for anergic disease,* Lb* infection leads to cellular hypersensitivity. To better understand the dichotomy in the immune response caused by these* Leishmania* species, we evaluated subsets of dendritic cells (DCs) and T lymphocyte in draining lymph nodes during the course of* La* and* Lb* infection in BALB/c mice. Our results demonstrated a high involvement of DCs in* La* infection, which was characterized by the greater accumulation of Langerhans cells (LCs); conversely,* Lb* infection led to an increase in dermal DCs (dDCs) throughout the infection. Considering the T lymphocyte response, an increase of effector, activated, and memory CD4^+^ T-cells was observed in* Lb* infection. Interleukin- (IL-) 4- and IL-10-producing CD4^+^and CD8^+^ T-cells were present in both* La* and* Lb* infection; however, interferon- (IFN-) *γ*-producing CD4^+^and CD8^+^ T-cells were detected only in* Lb* infection. The results suggest that during* Lb *infection, the dDCs were the predominant subset of DCs that in turn was associated with the development of Th1 immune response; in contrast* La* infection was associated with a preferential accumulation of LCs and total blockage of the development of Th1 immune response.

## 1. Introduction

American cutaneous leishmaniasis (ACL) is an anthropozoonosis transmitted by sand fly bites and caused by different species of the genus* Leishmania *in the New World. In Brazil, there are seven species that are recognized as causative agents of human diseases, but* Leishmania (V.) braziliensis* (*Lb*) and* L. (L.) amazonensis* (*La*) are considered the most medically important due to their higher incidence and disease severity [1].

There are different clinical forms of human* La* infection, varying from the localized cutaneous leishmaniasis (LCL) with moderate cellular hypersensitivity to anergic diffuse cutaneous leishmaniasis (ADCL), a cellular hyposensitivity pole of infection with a marked Th2-type immune response. Between the moderate LCL and the low-responsive ADCL, there is a weak-definite cellular hypersensitivity form known as borderline disseminated cutaneous leishmaniasis (BDCL), which has been shown to involve less immunosuppression than ADCL. On the other hand,* Lb *infection can cause not only LCL and BDCL but also mucocutaneous leishmaniasis (MCL), the cellular hypersensitivity pole of infection with a prominent Th1-type immune response [1, 2]. In this way, the ACL caused by these two* Leishmania* species presents a clinical-immunological spectrum where* La* shows a tendency to lead infection to the anergic pole of cellular immune response, whereas* Lb* leads infection to the hypersensitivity pole of host cellular immune response. The diversity of clinical manifestations has mainly been associated with antigenic differences of the different species of parasites [1], but also with the host immunogenetic background [3, 4].

Dendritic cells (DCs) are the most capable antigen-presenting cells (APC) and they possess the strong T-cell stimulatory capacity [5]. Langerhans cells (LCs) and dermal cells (dDCs) constitute major sentinel APC populations that reside in the skin [5–7]. Langerin (CD207) is a C-type lectin that is expressed on LCs, and the dermis contains two langerin^+^ DCs (distinguished by differential CD103 expression) and two subsets of langerin^−^ dDCs that differ in CD11b expression [8, 9].

Some studies have shown that in experimental* L. major* infection, the dDCs (langerin^−^) were able to stimulate antigen-specific T-cell proliferation, suggesting that dDCs are crucial for initiating an appropriate and effective cellular immune response [10], while LCs (langerin^+^) are not, as was previously postulated [11, 12]. In this way, Brewig et al. (2009) [13] reported that the priming of CD4^+^ T-cells was mediated by langerin^−^ dDCs, while langerin^+^ DCs were involved in the early priming of CD8^+^ T-cells, leading to parasite elimination in murine cutaneous leishmaniasis by* L. major*. Recently, working with parasites from the New World, we reported in the murine model of infection an increase in DC populations (CD207^+^ and CD11c^+^) and in T-cells (CD4^+^ and CD8^+^) at the dermal site of* L. (V.) braziliensis* infection. We also observed an increase in interferon- (IFN-) *γ* levels in draining lymph node (DLN) cells in relation to* L. (L.) amazonensis *infection, demonstrating the dichotomy in the immune response between these parasite species [14].

However, studies of the interaction between New World* Leishmania* species with innate and acquired immunities* in vivo *are not fully elucidated, reinforcing the importance of studying these parasite species in triggering the cellular immune response. As such, the main aim of this study was to analyze the influence of* La *and* Lb *on the phenotype of DCs in the DLNs of BALB/c mice, as well as investigate the relationship of DC subsets with the development of the T-cell immune response.

## 2. Materials and Methods

### 2.1. Mice

Eight-week-old BALB/c mice obtained from the Animal Facility of the São Paulo University, Medical School, Brazil, were maintained in our laboratory during the experiments, according to the guidelines of the institutional review board regarding the welfare of experimental animals, and with the approval of the Animal Ethics Committee of São Paulo University (protocol number: 0589/08).

### 2.2. Parasites

*Leishmania (Leishmania) amazonensis *(*La)* (MHOM/BR/1973/M2269) and* Leishmania (Viannia) braziliensis (Lb)* (MHOM/BR/1995/M15280) were kindly donated by Professor Fernando Tobias Silveira from Evandro Chagas Institute, Pará, Brazil.* La* and* Lb* parasites were isolated from patients with anergic diffuse cutaneous leishmaniasis and mucocutaneous leishmaniasis, respectively, in Pará state, northern Brazil. The parasites were grown in Schneider's Drosophila medium (Sigma-Aldrich Co., St. Louis, MO, USA), supplemented with 10% heat-inactivated fetal bovine serum (Gibco®; Thermo Fisher Scientific, Waltham, MA, USA), 10 *μ*g/mL of gentamicin, and 100 U/mL of penicillin at 25°C. On the 6th day of culture, promastigote forms from the stationary phase of culture growth were centrifuged at 1,670 g for 10 minutes using phosphate buffer saline (PBS) solution, pH 7.4, and subsequently used for the infection of mice.

### 2.3. Production of Whole Antigen of* La* and* Lb* Parasites

Promastigote forms of* La* and* Lb* (10^9^ promastigotes) in the stationary phase of growth were recovered by centrifugation at 1,200 g for 10 min at 4°C, followed by 3 washes with PBS at 1,200 g for 10 min at 4°C. Lysis buffer (20 mM Tris-HCl; 40 mM NaCl; 10 mM EDTA; 1% protease inhibitors cocktail) was added to the promastigote pellets and the material was frozen in liquid nitrogen and then thawed at room temperature three times to produce whole parasite antigens. Protein concentrations were estimated using the Bradford method.

### 2.4. BALB/c Mouse Infection

BALB/c mice were subcutaneously infected into the hind footpad with 10^6^ promastigote forms of* La* or* Lb* in the stationary phase from a low* in vitro* passage (≤6 passages) in 50 *μ*L of PBS. The control groups were injected with PBS.* La*,* Lb,* and control groups were composed of six mice each. In order to prove the evolution of infection, footpad swelling was measured weekly until 8 weeks postinfection (PI). At 4 and 8 weeks PI, popliteal DLNs were collected from the infected and control mice to determine parasite load and the phenotype of dendritic and T-cell subsets. A pool of popliteal lymph nodes from each group was prepared in triplicate to determine cell population diversity. Each experiment was independently repeated four times.

### 2.5. Analysis of the Parasite Load

The parasite load in the DLNs was determined via quantitative limiting-dilution assay, as described previously [15]. Briefly, the DLNs were aseptically excised and homogenized in Schneider's medium. The cellular suspension was subjected to 12 serial dilutions with four replicate wells. The number of viable parasites was determined from the highest dilution that promastigotes could be grown after 10 days of incubation at 25°C.

### 2.6. Flow Cytometry Assays

#### 2.6.1. Dendritic Cell Phenotyping

DLNs from* La- *and* Lb-*infected mice, as well as control animals, were aseptically collected, processed, and plated at 10^6^ cells/well in triplicate in 96-well V-bottomed plates in 100 *μ*L of FACS buffer (PBS with 0.5% bovine serum albumin and 2 mM of EDTA). The single-cell suspension was surface-stained with the following mouse monoclonal antibodies: anti-CD11c (clone N418), anti-CD11b (clone M1/70), anti-CD8*α* (53-6.7), anti-MHCII (clone 14-4-4S), anti-CD205 (clone 205yekta), anti-CD80 (clone 16-10A1), and anti-CD86 (clone GL1) at 4°C in the dark for 30 minutes. All antibodies were from BD Biosciences (Franklin Lakes, NJ, USA). The plates were then washed twice in flow cytometry buffer, and the cells were resuspended in 100 *μ*L of fixation buffer (1% paraformaldehyde in PBS; pH 7.5). The gating strategy used to determine the DC subsets is described in Supplementary Figure 1 in Supplementary Material available online at http://dx.doi.org/10.1155/2016/7068287.

#### 2.6.2. *In Vitro* Stimulation of T-Cells

The single-cell suspensions of DLN from* La*- and* Lb*-infected mice (10^6^ cells/well) were incubated in 96-well V-bottomed plates in the presence of 15 *μ*g/well of whole antigens of* La* and* Lb* parasites, respectively, in (RPMI)-1640 supplemented with 10% FBS, 2 mM of L-glutamine (Sigma-Aldrich Co.), 10 mM of Hepes buffer (Sigma-Aldrich Co.), 1 mM of sodium pyruvate, 1% vol/vol nonessential amino acid solution, 10 *μ*g/mL of gentamicin, and 1,000 U/mL of penicillin and 5 × 10^−5 ^M 2 *β*-mercaptoethanol (Sigma-Aldrich Co.) at 37°C in CO_2_ for 24 hours. The single-cell suspensions from the control mice were separately incubated with* La* or* Lb* antigens. Concanavalin A (1 *μ*g/well) was used as a positive control. 1 *μ*L/well of GolgiStop (BD Biosciences) was added, and cells were incubated for additional 16 hours (for IFN-*γ* and interleukin- [IL-] 10) and 24 hours (for IL-4). Plates were washed three times with FACS buffer and cells were surface-stained with the following mouse monoclonal antibodies: anti-CD3*ε* (clone 145-2C11; BD Biosciences), anti-CD4 (clone RM4-5; BD Biosciences), and anti-CD8*α* (clone 53-6.7; BD Biosciences) at 4°C in the dark for 30 minutes. Surface-stained cells were fixed and permeabilized using the Cytofix/Cytoperm*™* kit (BD Biosciences). Permeabilized cells were washed with Perm/Wash buffer (BD Biosciences) and stained with the following mouse monoclonal antibodies: anti-IFN-*γ* (clone XMG1.2; BD Biosciences), anti-IL-4 (clone 11B11; BD Biosciences), and anti-IL-10 (clone JES5-16E3; BD Biosciences) at 4°C in the dark for 30 minutes. The gating strategy used to determine the cytokine-producing T-cells is detailed in Supplementary Figure 2.

#### 2.6.3. Regulatory T Lymphocyte Phenotyping

Single-cell suspensions of DLN from* La- *and* Lb-*infected mice, as well as from control animals (10^6^ cells/well), were surface-stained with mouse monoclonal antibodies anti-CD4 (clone RM4-5; BD Biosciences) and anti-CD25 (clone PC61.5; BD Biosciences) at 4°C in the dark for 30 minutes. Cells were fixed and permeabilized as described above, and intracellular staining was carried out using mouse monoclonal antibody antiFoxP3 (clone FJK-16s; BD Biosciences) at 4°C in the dark for 30 minutes. The gating strategy used to determine regulatory T-cell subsets is described in Supplementary Figure 2.

#### 2.6.4. Memory T Lymphocyte Phenotyping

Single-cell suspensions of DLN from* La- *and* Lb-*infected mice, as well as from control animals (10^6^ cells/well), were surface-stained at 4°C in the dark for 30 minutes with the following mouse monoclonal antibodies: anti-CD3*ε* (clone 145 2C11; BD Biosciences), anti-CD4 (clone RM45; BD Biosciences), anti-CD8*α* (clone 53-6.7; BD Biosciences), anti-CD45RB (clone16A; BD Biosciences), and anti-CD62L (clone MEL14; BD Biosciences). The gating strategy used to determine memory T-cell subsets is described in Supplementary Figure 2.

All samples were acquired on FACSFortessa using FACSDiva software (BD Biosciences), and they were then analyzed with FlowJo software version 9.2 (Tree Star; FlowJo, LLC, Ashland, OR, USA). Fluorescence voltages were determined using matched unstained cells. Compensation was carried out with the aid of CompBeads (BD Biosciences) single stained with the monoclonal antibodies described earlier. Samples were acquired to reach at least 300,000 events.

### 2.7. Statistical Analysis

Prism 5.00 for Windows (GraphPad Software Inc., La Jolla, CA, USA) was used to perform all statistical analyses. Differences between the experimental groups were analyzed by the nonparametric Mann-Whitney* U* test. Correlations between cellular populations were analyzed by Spearman's correlation coefficient. A *p* value < 0.05 was considered significant.

## 3. Results

### 3.1. *La* Infection Leads to Higher Cutaneous Lesion Size and Parasite Load in DLN

**Figure 1:**
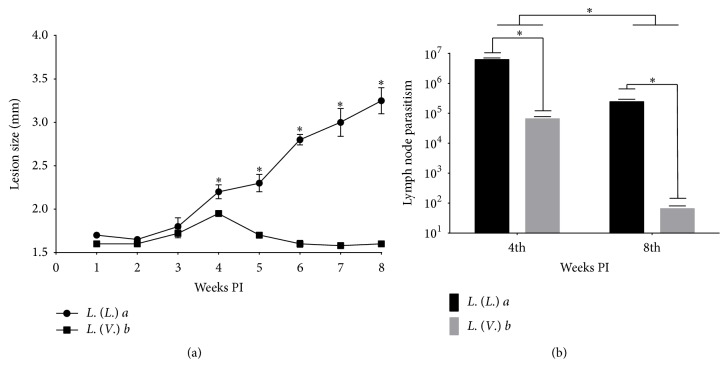
*L. (L.) amazonensis* and* L. (V.) braziliensis* infection in BALB/c mice. (a) The evolution of the lesion size (mm) in the hind footpad of mice infected with* L. (L.) amazonensis* or* L. (V.) braziliensis*. (b) Parasite load (number of parasites/mg of tissue) in the draining lymph nodes of mice infected with* L. (L.) amazonensis* or* L. (V.) braziliensis*. Each data point represents the mean ± standard deviation for the respective experimental group. ^*∗*^ *p* < 0.05.

As previously described by our group [14],* La* infection induced a progressive growth of skin lesions in BALB/c mice from 3 weeks PI accompanied by high tissue parasitism (data not shown), and it was much higher than the lesion caused by* Lb *during the evolution of the infection. Conversely,* Lb* infection induced mild hind footpad swelling between 3 and 5 weeks PI with regression from week 6 PI (Figure 1(a)).

In the draining lymph nodes, the parasite load in* La* infection was higher than in* Lb *infection at 4 and 8 weeks PI. From 4 to 8 weeks PI, the number of parasites decreased in both infections; however, in* Lb* infection the parasitism decreased by 1000-fold while in* La* infection there was a 25-fold decrease (Figure 1(b)).

### 3.2. *La* Infection Showed a Higher Number of CD11c^+^MHCII^+^CD80^+^ and CD11c^+^MHCII^+^CD86^+^ Cells Than* Lb* Infection

**Figure 2:**
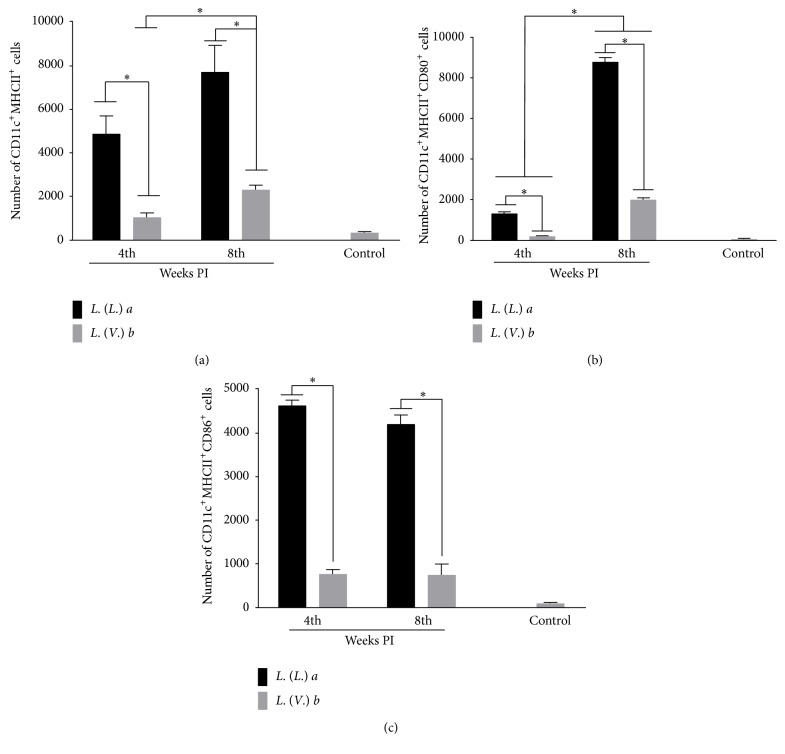
Phenotyping dendritic cells (DCs) from the popliteal lymph nodes of BALB/c mice infected with* L. (L.) amazonensis* or* L. (V.) braziliensis* and their activation. (a) DCs were stained for the surface expression of CD11c and MHC class II molecules, (b) and their status of activation was analyzed using CD80 (b) and CD86 (c) markers. Data represent the mean ± SD of three independent experiments. ^*∗*^ *p* < 0.05.

The number of CD11c^+^MHCII^+^ cells in DLN which include different subsets of DCs was significantly higher in* La* than in* Lb* infection at 4 and 8 weeks PI. However, during the evolution of the* La* infection, no difference was observed in the number of CD11c^+^MHCII^+^ cells, while in the evolution of the* Lb* infection, a significant increase was observed (Figure 2(a)).

The activation of these cells was analyzed by staining the costimulatory molecules, CD80 and CD86, on the surface of CD11c^+^MHCII^+^ cells. In this regard, BALB/c mice infected with* La* presented a higher number of CD11c^+^MHCII^+^CD80^+^ cells compared to* Lb* infection at 4 and 8 weeks PI. A significant increase in the number of this cell subset was observed in both* La* and* Lb *during the evolution of the infection (Figure 2(b)). Similarly, the number of CD11c^+^MHCII^+^CD86^+^ cells was higher in* La* infection compared to* Lb* infection at 4 and 8 weeks PI. However, during the course of infection, the number of CD11c^+^MHCII^+^CD86^+^ cells did not change in either infection (Figure 2(c)).

### 3.3. *La* Infection Induced an Increase in LCs, While* Lb* Infection Increased dDCs

**Figure 3:**
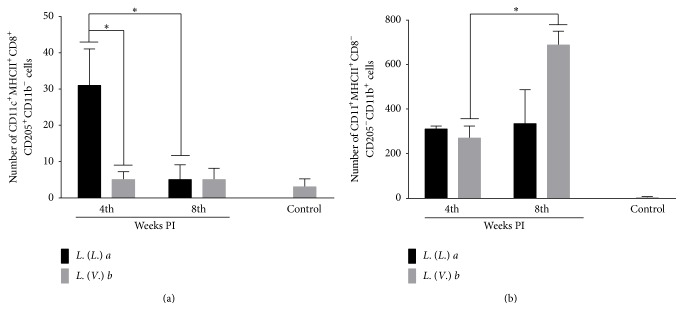
Phenotyping of dendritic cell subsets from the popliteal lymph nodes of BALB/c mice infected with* L. (L.) amazonensis* or* L. (V.) braziliensis*. (a) Langerhans cells (CD11c^+^MHC^+^CD8^+^CD205^+^CD11b^−^) and (b) dermal dendritic cells (CD11c^+^MHC^+^CD8^−^CD205^−^CD11b^+^) were phenotyped at the 4th and 8th week postinfection. Data represent the mean ± SD of three independent experiments. ^*∗*^ *p* < 0.05.

The number of LCs (CD11c^+^MHC^+^CD8^+^CD205^+^CD11b^−^) was significantly higher in the* La* infection than in the* Lb* infection at 4 weeks PI. However, at 8 weeks PI, no difference was observed between* La* and* Lb* infection. During the evolution of infection, the number of LCs significantly decreased in* La* infection, but it did not change in* Lb *infection (Figure 3(a)). The number of dDCs (CD11c^+^MHC^+^CD8^−^CD205^−^CD11b^+^) was similar between* La* and* Lb* infection at 4 weeks PI, but it was higher in* Lb* infection at 8 weeks PI. In relation to the evolution of infection, the number of dDCs increased during* Lb* infection, but not during* La* infection (Figure 3(b)).

### 3.4. *Lb* Infection Showed Evidence of Effector, Activated, and Memory CD4^+^ T-Cells

**Figure 4:**
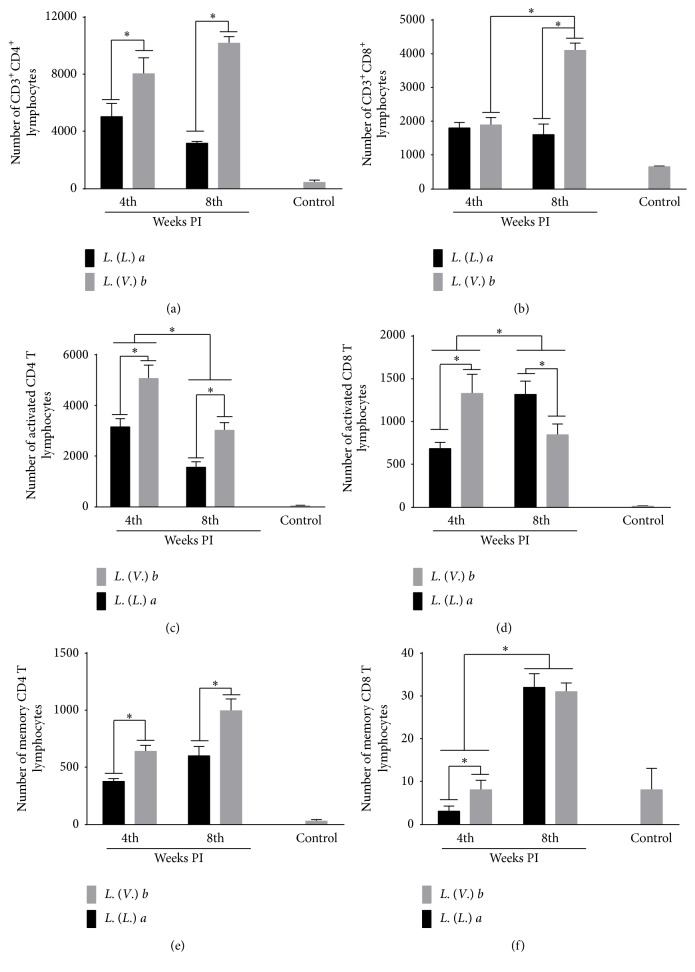
CD4^+^ and CD8^+^ T-cell immunophenotyping from the popliteal lymph nodes of BALB/c mice infected with* L. (L.) amazonensis* or* L. (V.) braziliensis*. (a) Number of CD3^+^CD4^+^ T-cells. (b) Number of CD3^+^CD8^+^ T-cells. (c) Number of activated CD4^+^ T-cells (CD3^+^CD4^+^CD62L^low^CD45RB^high^). (d) Number of activated CD8^+^ T-cells (CD3^+^CD8^+^CD62L^low^CD45RB^high^). (e) Number of memory CD4^+^ T-cells (CD3^+^CD4^+^CD62L^low^CD45RB^low^). (f) Number of memory CD8^+^ T-cells (CD3^+^CD8^+^CD62L^low^CD45RB^low^). Data represent the mean ± SD of three independent experiments. ^*∗*^ *p* < 0.05.

The number of CD4^+^, activated (CD3^+^CD4^+^CD62L^low^CD45RB^high^), and memory (CD3^+^CD4^+^CD62L^low^CD45RB^low^) T-cells in the DLN was higher in* Lb *infection than in* La* infection at 4 and 8 weeks PI (Figures 4(a), 4(c), and 4(e), resp.). However, the number of activated CD4^+^ T-cells decreased during the evolution of* Lb* and* La* infection.

The number of CD8^+^ T lymphocytes in the DLN was similar during* La* and* Lb* infection at 4 weeks PI, although, at 8 weeks PI, the number of CD8^+^ T-cells was higher in the* Lb* than in the* La* infection (Figure 4(b)). The number of activated CD8^+^ T lymphocytes was higher in* Lb* infection when compared to* La *infection at 4 weeks PI, but the number of activated CD8^+^ T-cells increased in* La* infection and decreased in* Lb* infection throughout the course of the infection (Figure 4(d)). The number of memory CD8^+^ T-cells was higher in* Lb* than in* La* infection at 4 weeks PI; however, at 8 weeks PI, the number of CD8^+^ memory T lymphocytes was similar between* Lb* and* La* infection. Concerning the evolution of the infection, the number of CD8^+^ memory T lymphocytes increased in both infections (Figure 4(f)).

### 3.5. IFN-*γ*-Producing CD4^+^ and CD8^+^ T-Cells Were Detected Only in* Lb* Infection

**Figure 5:**
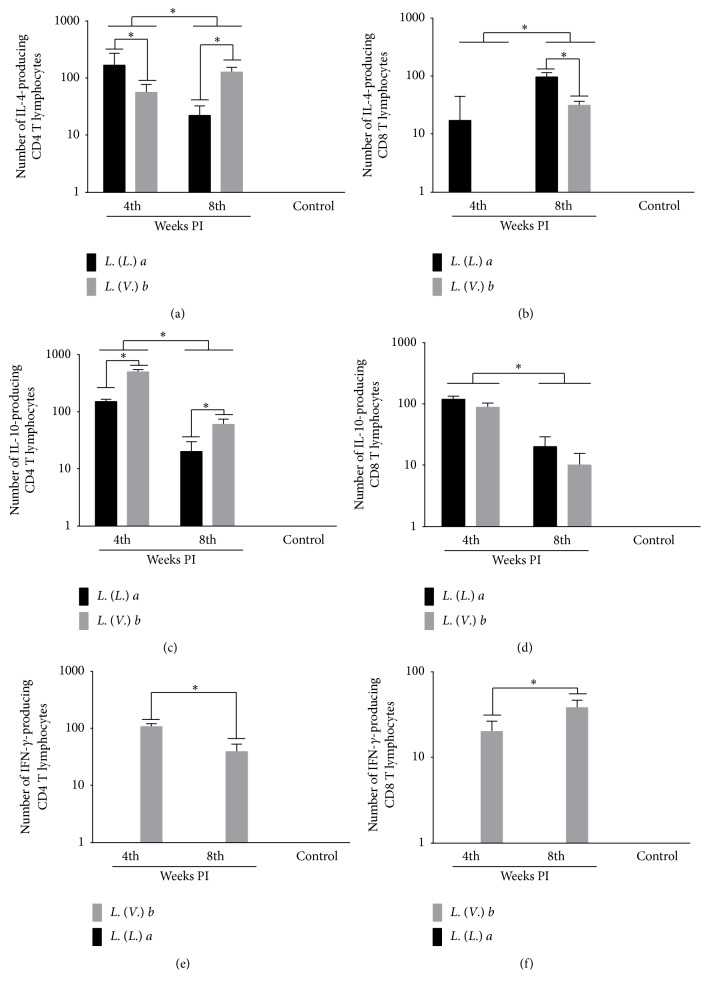
IL-4-, IL-10-, and IFN-*γ*-producing CD4^+^ and CD8^+^ T-cells from the popliteal lymph nodes of BALB/c mice infected with* L. (L.) amazonensis* or* L. (V.) braziliensis*. (a) Number of IL-4-producing CD4^+^ T-cells. (b) Number of IL-4-producing CD8^+^ T-cells. (c) Number of IL-10-producing CD4^+^ T-cells. (d) Number of IL-10-producing CD8^+^ T-cells. (e) Number of IFN-*γ*-producing CD4^+^ T-cells. (f) Number of IFN-*γ*-producing CD8^+^ T-cells. Data represent the mean ± SD of three independent experiments. ^*∗*^ *p* < 0.05.

*La *infection showed a higher number of the IL-4-producing CD4^+^ T lymphocyte subset than* Lb* infection at 4 weeks PI; however, at 8 weeks PI,* La *infection showed a lower number of IL-4-producing CD4^+^ T-cells than* Lb *infection. During the evolution of the infection, the population of IL-4-producing CD4^+^ T-cells increased during the* Lb* infection and decreased during the* La *infection (Figure 5(a)). IL-4-producing CD8^+^ T-cells were detected only during the* La* infection at 4 weeks PI, but it was present in both infections at 8 weeks PI; moreover, it was higher in the* La* than in the* Lb* infection. During the evolution of the infection, the number of IL-4-producing CD8^+^ T-cells increased in both infections (Figure 5(b)).

The number of IL-10-producing CD4^+^ T-cells was higher in the* Lb* than in the* La* infection at 4 and 8 weeks PI. During the evolution of the infection, the number of IL-10-producing CD4^+^ T-cells decreased in both infections (Figure 5(c)). On the other hand, the number of IL-10-producing CD8^+^ T-cells was similar between the* La* and* Lb* infection at 4 and 8 weeks PI, and it decreased throughout the evolution of the infection (Figure 5(d)).

Of note, the number of IFN-*γ*-producing CD4^+^ and CD8^+^ T-cells was detected only in the* Lb* infection; however, on the evolution of the infection, the number of IFN-*γ*-producing CD4^+^ T-cells decreased, while the number of IFN-*γ*-producing CD8^+^ T-cells increased (Figures 5(e) and 5(f)).

### 3.6. *Lb* Infection Induces Natural and Inducible Treg Cells

**Figure 6:**
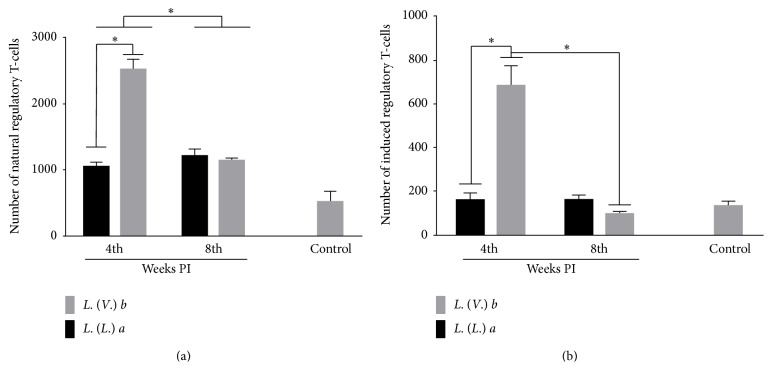
Phenotyping of subsets of regulatory T-cells in the popliteal lymph nodes of BALB/c mice infected with* L. (L.) amazonensis* or* L. (V.) braziliensis*. (a) Natural regulatory T-cells (CD4^+^CD25^+^FOXP3^+^). (b) Inducible regulatory T-cells (CD4^+^FOXP3^+^). Data represent the mean ± SD of three independent experiments. ^*∗*^ *p* < 0.05.

The number of natural regulatory T-cells (CD4^+^CD25^+^FoxP3^+^) was higher in* Lb *than in* La* infection at 4 weeks PI; however, at 8 weeks PI, the number of natural Treg cells was similar in* Lb* and* La* infection. Nevertheless, during the evolution of the infection, this lymphocyte subset decreased in animals infected with* Lb*, while it was sustained throughout the course of the* La *infection (Figure 6(a)). The number of induced Treg cells (CD4^+^Foxp3^+^) was higher in the* Lb* than in the* La* infection at 4 weeks PI but decreased in the DLN of mice infected with* Lb* during the evolution of the infection (Figure 6(b)).

## 4. Discussion

The immunological response in cutaneous leishmaniasis occurs in the vicinity of the primary sites of infection, mainly in draining lymph nodes. At these sites, parasites proliferate inside phagocytic cells and alter the physiology and morphology of lymphoid organs. These effects impact the cells of the innate and acquired immune responses, and consequently the outcome of infection. In the present study, it was demonstrated that the clinical response induced by* La* was associated with progressive disease, as evaluated by lesion size and parasitism; in contrast,* Lb* induced a self-healing lesion with low parasitism. Therefore, these parasites induced distinct clinical outcomes in BALB/c mice that were associated with number, subsets, and status of activation of DCs and T lymphocytes.

In this study, it was shown that lymph nodes of BALB/c mice infected with* La* accumulated more DCs characterized by CD11c^+^MHCII^+^ phenotype compared to* Lb *infection. This was associated with an early increase in the number of Langerhans cells in* La* infection, and a late increase in the number of dDC in* Lb* infection. In human American cutaneous leishmaniasis, the presence of Langerhans cells has been associated with infecting parasite species and gravity of disease, since patients infected with* La* presented higher numbers of Langerhans cells in the epidermis compared to those infected with* Leishmania (Viannia) *sp. In addition,* La*-infected patients were not able to stimulate a proper cellular immune response, as shown by a low accumulation of T-cells in the skin [16]. However, it has been demonstrated that dDCs are able to efficiently stimulate cellular immune responses in experimental cutaneous leishmaniasis, suggesting that dDCs are crucial to the initiation of a specific T lymphocyte response [10]. Thus, these results suggest that the accumulation of Langerhans cells in the lymph nodes of BALB/c mice during* La* infection is associated with disease progression, while a late accumulation of dDCs in the lymph nodes of BALB/c mice infected with* Lb* is related to a more favorable prognosis in these animals, as evidenced by the mounting of self-healing process and a decrease in the number of parasites.

Indeed, during the* Lb *infection, accumulation of dDCs had a direct correlation with the presence of CD8^+^ T-cells, and an inverse correlation with IL-10-producing CD4^+^ and -CD8^+^ T-cells, suggesting that dDCs are crucial to induce an efficient priming of T lymphocytes. On the other hand, LCs (MHCII^+^CD11c^+^CD8^+^CD205^+^CD11b^−^) were also detected in the lymph nodes of BALB/c mice during the course of* Lb* infection, but at a similar number as those in control mice and in lesser amount compared with* La* infection, suggesting that LCs have a less prominent role or perhaps no role in the pathogenesis of* Lb* infection. On the other hand, during* La* infection, the LC population increased at early stages of infection compared to* Lb* infection, with a positive correlation with IL-4-producing CD4^+^ T-cells and an inverse correlation with IFN-*γ*-producing CD4^+^ T-cells, consequently aiding development of a Th2 response and disease progression.

Previously, our group demonstrated an increase in cell densities of both DC populations (CD11c^+^ and CD207^+^) in the skin of BALB/c mice infected with* La* when compared to those infected with* Lb*. Moreover, the levels of IFN-*γ* were higher only in the* Lb* infection, but the levels of IL-4 and IL-10 were higher in the* La* infection [14]. Thus, the retention of dDCs in the lymphoid organs of animals infected with* Lb* suggest that the presence and accumulation of a high number of dDCs in lymph nodes in the late phases of infection can trigger an immunological response associated with parasite elimination. In contrast, animals with progressive disease caused by* La* were not able to retain dDCs in the lymphoid organs and yet presented with a decreased Th1 populations, suggesting that dDCs may trigger a beneficial response to the host [17].

There is general agreement that DCs are among the most potent APCs to stimulate primary T-cell activation. It is also known that the type and strength of costimulatory signals delivered by DCs to T-cells can affect the development of distinct lymphocyte subsets [18]. Brown et al. (1996) [19] reported that the blockade of CD86, but not CD80, in mice susceptible to* L. major* infection led to a decrease in parasite burden and production of Th2 cytokines. These results suggest that CD86, and not CD80, is critical for Th2 differentiation. Studies have suggested that* La* was able to infect and alter DC biology, favoring the establishment of the infection. In addition, murine models have been used to demonstrate that* La* amastigotes and promastigotes infect and activate DCs; however, amastigote antigens fail to induce CD40-dependent IL-12 production, triggering a Th2 immune response, mediated mainly by IL-4 production [20]. Additionally, in spite of a decrease in CD80 expression, an increase in CD86 was observed in the* in vitro* interaction of* La* with human DCs and these phenotypic changes were accompanied by lower secretion of IL-6 and IFN-*γ* [21]. Corroborating these data, our study showed a high number of both CD80^+^ and CD86^+^ cells in* La* infection in animals that presented progressive skin lesions and parasite burden in skin [14] and popliteal lymph nodes. Moreover, these animals did not present IFN-*γ*-producing T lymphocytes, resulting in a susceptibility profile. By contrast, in* Lb* infection a low number of CD86-activated DCs was detected in popliteal lymph nodes, and this was associated with an effective immune response, mediated by IFN-*γ*, and, consequently, the number of parasites drastically decreased during the course of infection.

Infection with* La* or* Lb* induced an increase in CD4^+^ T-cells in DLN; however, the number of CD4^+^ T-cells was greater in the* Lb *infection and displayed a Th1 immune phenotype, since IFN*γ*-producing CD4^+^ T-cells were only observed in the infection with* Lb* and not during* La* infection. In spite of that, an increase in the number of IL-4- and IL-10-producing CD4^+^ T-cells was observed in BALB/c mice infected with* Lb*. Remarkably, Qi et al. [20] showed that the DLN cells of BALB/c mice infected with* La* may produce both Th1 (IFN-*γ*) and Th2 (IL-4 and IL-10) cytokines, and the magnitude of the Th2 response is linked to a high production of IL-4 and IL-10 cytokines, which are responsible for the establishment of* La* in experimental hosts. Moreover, our results showed that the number of activated CD4^+^ T-cells (CD62L^low^CD45RO^high^) was higher in* Lb *infection during the time course experiment. Corroborating these findings, it was further demonstrated that self-healing during* L. major* murine infection is associated with a protective Th1 response characterized by early IFN-*γ* production and the expression of inducible NO synthase by activated macrophages [22].

In addition, during* La* infection, a positive correlation was observed between the number of activated CD4^+^ T-cells and IL-4- and IL-10-producing CD4^+^ T-cells, but CD4^+^IFN-*γ* ^+^ T-cells were not stimulated. It is important to point out that* La *is responsible for causing anergic diffuse cutaneous leishmaniasis, a rare form of cutaneous disease, where patients present highly parasitized multiple lesions and lack IFN-*γ* production [2]. Similarly, the number of CD4^+^ memory cells (CD62L^low^CD45RO^low^) was higher in the DLN cells of the BALB/c mice infected with* Lb* at 8 weeks PI, while a positive correlation was maintained with the number of IFN-*γ*-producing CD4^+^ T-cells. Therefore, our results support the idea that* La *can survive in host cells due to its dampening of the hosts adaptive immune response, while the production of IFN-*γ* may be responsible for the control of tissue parasitism in* Lb* infection.

With regard to the role of CD8^+^ T-cells, it should be highlighted that these cells have been associated with healing and protection in human and murine leishmaniasis and that their activation is dependent on CD4^+^ T-cells and DC cells [23–26]. Our results showed that the participation of CD8^+^ T-cells in the DLN was less evident than the involvement of CD4^+^ T-cells during infection with both species of the parasite. However, the number of CD8^+^ T-cells was higher during* Lb* infection, mainly at the end of the time course experiment, which positively correlates with the number of IFN-*γ*-producing CD8^+^ T-cells. Interestingly, we also observed an increase of activated CD8^+^ T-cells (CD62L^low^CD45RO^high^) during infection with* La *compared to the control. Indeed, it has been shown that, in* Leishmania* infection, the protective role of CD8^+^ T-cells is most likely due to their ability to proliferate and produce large amounts of IFN-*γ* [25], as we observed in* Lb* infection.

An increase in the number of memory CD8^+^ T-cells (CD62L^low^CD45RO^low^) was evident at 8 weeks PI in both* La* and* Lb* infection, compared to the control. Interestingly, there was a direct correlation between these cells and IFN-*γ*-producing CD8^+^ T-cells, as well as an indirect correlation with IL-10 producing CD8^+^ T-cells in* Lb*, suggesting that these memory cells are connected to the protective response. Previous studies showed that CD8^+^ T-cells produce large amounts of IFN-*γ* following* L. major* infection and that these cells were critical for optimum primary immunity [27, 28].

Regulatory T- (Treg) cells play a critical role in the control of the immune response, including that induced by tumors, transplanted organs, and parasite antigens. Many studies with human experimental models of leishmaniasis have focused on the role of Treg cells in controlling tissue destruction at the sites of infection [29–32]. Several types of Treg cells have been described on the basis of their origin, generation, and mechanism of action, with two main subsets identified: naturally occurring CD4^+^CD25^+^ regulatory T-cells (nTReg cells) and inducible regulatory T-cells (iTReg). In addition, the expression of FoxP3, a transcription factor known to be crucial for the development and function of both nTReg and iTreg cells, was also evident [31, 33–35]. Our results highlighted that both populations of Treg cells (iTReg and nTReg) were present in higher numbers in the DLN of BALB/c mice infected with* Lb* at 4 weeks PI. nTReg cells were also observed at the outcome of* La* infection, though in low numbers. Similar findings were observed by Falcão et al. in 2012 [36]; this group reported a high frequency of Treg cells both in skin lesions and in the DLN during experimental infection caused by* Lb*. Interestingly, the number of nTReg and iTReg cells decreased throughout the course of* Lb* infection, which clearly correlates with a strong effector T-cell response, decreased lesion size, and low parasitic load in the DLN at 8 weeks PI. However, during* La* infection, nTReg cells showed a slight increase during the ongoing infection that coincided with an increase in lesion size and skin parasitism, as has already been shown [14]. Corroborating our results, a study conducted by Ji et al. [37] revealed a rapid accumulation of CD4^+^CD25^+^ Treg cells following infection with* La*, which correlated with the increased of TGF-*β*, FoxP3, and IL-10R in local tissues. However, this benevolent role is transitory, suggesting the importance of a fine balance between Treg and effector T-cells in regulating host susceptibility to* La* infection.

## 5. Concluding Remarks 

To the best of our knowledge this is the first study comparing the number, subsets, and activation status of DCs and T lymphocytes during* L. (L.) amazonensis* or* L. (V.) braziliensis* infections using the same genetic background of the vertebrate host.

In summary, the findings indicated that during* L. (V.) braziliensis* infection the dDCs were the dominant subset of DCs present in lymph nodes of BALB/c mice, which, in turn, were associated with the activation of both CD4 and CD8 T lymphocytes, which were able to produce IFN-*γ*. The presence of Treg lymphocytes in these animals can be associated with regulation of the immune system, in order to prevent tissue damage caused by an excessive immune response, as observed in* Lb* infection, although a role for persistence of the parasite in lymph nodes may also be attributed to these lymphocyte subsets. Therefore, the presence of dDCs was related to development of Th1 immune response and tissue parasitism control during* Lb* infection.

In contrast,* L. (L.) amazonensis* infection is associated with a preferential accumulation of LCs at 4 weeks PI in lymph nodes, and total blockage of the development of IFN-*γ*-producing CD4 and CD8 T lymphocytes, and thus a clear development of Th2 immune response. Therefore, LCs can be linked to parasite escape from the immune system, since antigen-specific Th1 cells were not clearly differentiated, contributing to the parasite persistence in experimental host.

The results obtained in this study add new knowledge to the complexity of the immune response against the two most important species of* Leishmania* that cause cutaneous disease in the New World.

## Supplementary Material

Supplementary Figure 1: Gating strategy used for all experiments of immunolabeling to determine dendritic cell subsets in lymph nodes of BALB/c mice infected with *L. (L.) amazonensis* or *L. (V.) braziliensis*.
Supplementary Figure 2: Gating strategy used to identify T lymphocyte subsets during experimental infections caused by *L. (L.) amazonensis* or *L. (V.) braziliensis*. (a) Identification of CD4+ and CD8+ T lymphocytes, (b) Identification of memory T lymphocytes, (c) Identification of regulatory T lymphocytes, (d) Identification of cytokine-producing T lymphocytes.
