# Supplementary figures and images for: Differential Recruitment of Dendritic Cells Subsets to Lymph Nodes Correlates with a Protective or Permissive T-Cell Response during Leishmania (Viannia) Braziliensis or Leishmania (Leishmania) Amazonensis Infection
Source: Mediators Inflamm. 2016 Mar 17;2016:7068287. doi: 10.1155/2016/7068287 (PMC4814687; doi:10.1155/2016/7068287)

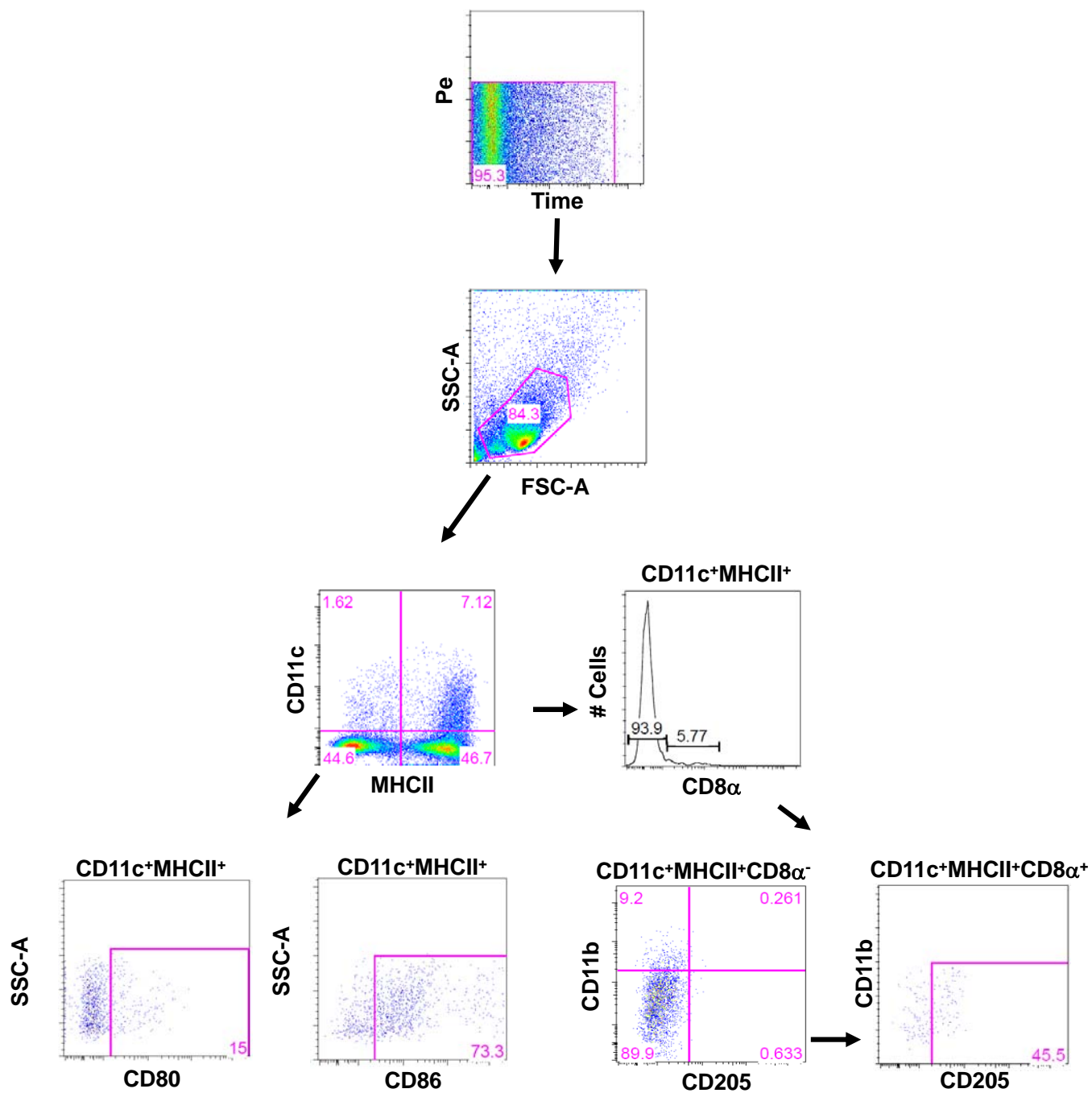

Figure 1

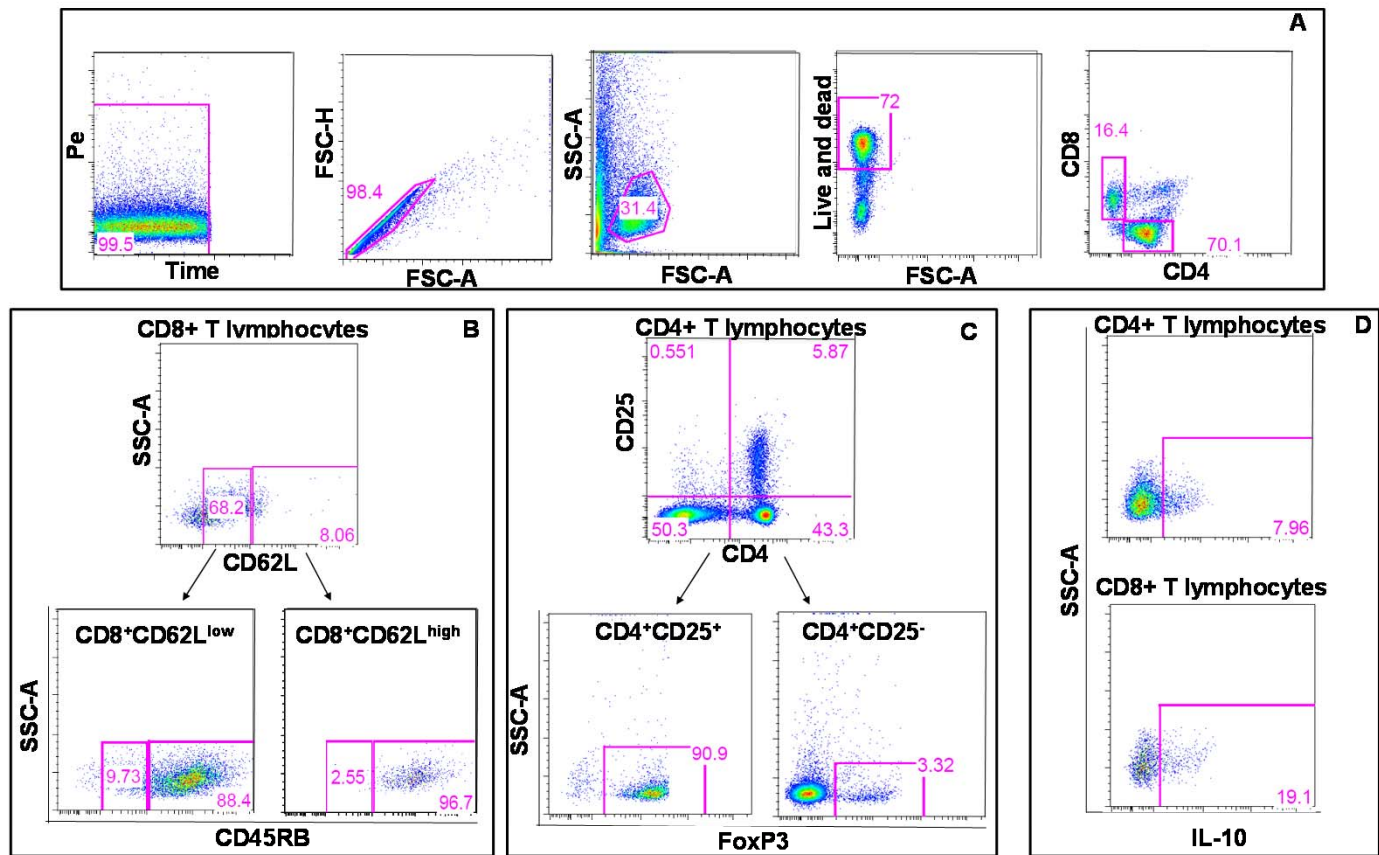

Figure 2

Supplement: Supplementary file 1 — Supplementary Figure 1: Gating strategy used for all experiments of immunolabeling to determine dendritic cell subsets in lymph nodes of BALB/c mice infected with L. (L.) amazonensis or L. (V.) braziliensis. Supplementary Figure 2: Gating strategy used to identify T lymphocyte subsets during experimental infections caused by L. (L.) amazonensis or L. (V.) braziliensis. (a) Identification of CD4+ and CD8+ T lymphocytes, (b) Identification of memory T lymphocytes, (c) Identification of regulatory T lymphocytes, (d) Identification of cytokine-producing T lymphocytes. [file 7068287.f1.pdf]
